# Supplementary material for: Two-qubit quantum gate and entanglement protected by circulant symmetry
Source: Sci Rep. 2020 Mar 19;10:5030. doi: 10.1038/s41598-020-61766-w (PMC7081313; doi:10.1038/s41598-020-61766-w)
Supplement: Supplementary file 1 — Supplementary Information. [file 41598_2020_61766_MOESM1_ESM.pdf]

Supplemental Material for "Two-qubit gate and entanglement protected by circulant symmetry"

Peter A. Ivanov[1] and Nikolay V. Vitanov[1]
[1]*Department of Physics, St. Kliment Ohridski University of Sofia, James Bourchier 5 blvd, 1164 Sofia, Bulgaria*

DERIVATION OF THE INSTANTANEOUS EIGENSPECTRUM

We consider the Hamiltonian

$$\hat{H}(t) = J(\hat{\sigma}_1^+ + \hat{\sigma}_1^-)(\hat{\sigma}_2^+ e^{-i\varphi} + \hat{\sigma}_2^- e^{i\varphi}) + \Omega_1(\hat{\sigma}_1^+ + \hat{\sigma}_1^-) + \Omega_2(\hat{\sigma}_2^+ e^{i\varphi} + \hat{\sigma}_2^- e^{-i\varphi}), \quad (1)$$

where the first term describes the spin-spin interaction with coupling J and the second two terms are the single spin drives with Rabi frequencies $\Omega_{1,2}$. The instantaneous eigenfrequencies are

$$\chi_{\pm}(t) = \Omega_1 \pm \sqrt{J^2 + \Omega_2^2 + 2J\Omega_2\cos(2\varphi)}, \quad \nu_{\pm}(t) = -\Omega_1 \pm \sqrt{J^2 + \Omega_2^2 + 2J\Omega_2\cos(2\varphi)} \quad (2)$$

In order to have a non-degenerate eigenfrequencies at the initial moment we require that $\Omega_1(t_{\rm i}) \neq \Omega_2(t_{\rm i})$. For simplicity we set $\varphi = \pi/4$ and the corresponding eigenvectors are

$$|\chi_+(t)\rangle = \frac{1}{2}\{e^{-i\frac{\pi}{4}+i\xi(t)}|\downarrow\downarrow\rangle + |\downarrow\uparrow\rangle + e^{-i\frac{\pi}{4}+i\xi(t)}|\uparrow\downarrow\rangle + |\uparrow\uparrow\rangle\}$$
$$|\chi_-(t)\rangle = \frac{1}{2}\{e^{-i\frac{\pi}{4}+i\xi(t)}|\downarrow\downarrow\rangle - |\downarrow\uparrow\rangle + e^{-i\frac{\pi}{4}+i\xi(t)}|\uparrow\downarrow\rangle - |\uparrow\uparrow\rangle\} \quad (3)$$

and

$$|\nu_+(t)\rangle = \frac{1}{2}\{e^{i\frac{\pi}{4}-i\xi(t)}|\downarrow\downarrow\rangle + i|\downarrow\uparrow\rangle - e^{i\frac{\pi}{4}-i\xi(t)}|\uparrow\downarrow\rangle - i|\uparrow\uparrow\rangle\},$$
$$|\nu_-(t)\rangle = \frac{1}{2}\{e^{i\frac{\pi}{4}-i\xi(t)}|\downarrow\downarrow\rangle - i|\downarrow\uparrow\rangle - e^{i\frac{\pi}{4}-i\xi(t)}|\uparrow\downarrow\rangle + i|\uparrow\uparrow\rangle\}. \quad (4)$$

where we define

$$\tan\xi(t) = \frac{\Omega_2}{J}. \quad (5)$$

Adiabatic following the instantaneous eigenstates implies the following set of transitions:

$$|{-}{-}\rangle \to e^{i\alpha}|\psi_3\rangle,$$
$$|{-}{+}\rangle \to e^{-i\beta}|\psi_1\rangle,$$
$$|{+}{-}\rangle \to e^{i\beta}|\psi_2\rangle,$$
$$|{+}{+}\rangle \to e^{-i\alpha}|\psi_0\rangle, \quad (6)$$

where $|\pm_1\rangle = (|\downarrow_1\rangle \pm |\uparrow_1\rangle)/\sqrt{2}$ and $|\pm_2\rangle = (|\downarrow_2\rangle \pm e^{i\varphi}|\uparrow_2\rangle)/\sqrt{2}$ are the initial rotating computational spin states and $\alpha = \int_{t_{\rm i}}^{t_{\rm f}} \chi_+(t)dt$, $\beta = \int_{t_{\rm i}}^{t_{\rm f}} \nu_+(t)dt$ are the adiabatic phases. We choose the following time-dependence of the couplings

$$J(t) = J_0\sin^2(\omega t), \quad \Omega_1(t) = \Omega_1, \quad \Omega_2(t) = J_0 + V_0\cos^2(\omega t), \quad (7)$$

which can be used to implement the set of transitions (6). Here the interaction time vary in the interval $t \in [0, t_{\max}]$ with $t_{\max} = \pi/(2\omega)$. For given value of J_0 and ω one can find Ω_1 and V_0 such that the adiabatic phases becomes $\alpha = 2k\pi$, $\beta = 2p\pi$. As a figure of merit for the fidelity of the adiabatic transitions (6) we use (see Fig. 4(b) in the main text)

$$F(t) = \frac{1}{16}|\langle\psi_0|\chi_+(t)\rangle + \langle\psi_1|\nu_+(t)\rangle + \langle\psi_2|\chi_-(t)\rangle + \langle\psi_3|\nu_-(t)\rangle|^2. \quad (8)$$
